# Mitochondrial disturbance related to increased caspase-1 of CD4^+^T cells in HIV-1 infection

**DOI:** 10.1186/s12879-023-08485-5

**Published:** 2024-01-24

**Authors:** Fengting Yu, Chengjie Ma, Xia Jin, Hongxin Zhao, Jiang Xiao, Li Li, Shujing Song, Xiaohui Xie, Siyuan Yang, Yunxia Tang, Linghang Wang, Fujie Zhang

**Affiliations:** 1 Medical School, University of Chinese Academy of Sciences, Beijing, 101400 China; 2 Beijing Ditan Hospital, Capital Medical University, Beijing, China; 3 Clinical Center for HIV/AIDS, Capital Medical University, Beijing, China; 4 Human Viral Diseases and Vaccine Translation Research Unit, Key Laboratory of Molecular Virology & Immunology, Institut Pasteur of Shanghai, Chinese Academy of Sciences, Shanghai, China; 5 Department of Infectious Diseases, Peking University Ditan Teaching, Hospital, Beijing, China

**Keywords:** HIV/AIDS, CD4^+^T pyroptosis, Mitochondrial mass, MFN1

## Abstract

**Background:**

In HIV-1 infection, more than 95% of CD4^+^T cells die of caspase-1 mediated pyroptosis. What governs the increased susceptibility of CD4^+^T cells to pyroptosis is poorly understood.

**Methods:**

Blood samples were obtained from 31 untreated HIV-infected patients (UNT), 29 antiretroviral therapy treated HIV-infected patients (ART), and 21 healthy control donors (HD). Plasma levels of IL-18 and IL-1β, caspase-1 expression, mitochondrial mass (MM) and mitochondrial fusion/fisson genes of CD4^+^T subsets were measured.

**Results:**

A significantly higher IL-18 level in plasma and MM level of CD4^+^T cells were found in HIV-infected patients (UNT and ART) compared to HD, and the MM^high^ phenotype was manifested, related to increased caspase-1 expression. Moreover, the increased MM was more pronounced in the early differentiated and inactivated CD4^+^T cells. However, higher MM was not intrinsically linked to T cell differentiation disorder or excessive activation of the CD4^+^T cells. Mechanistically, the increased MM was significantly correlated with an elevated level of expression of the mitochondrial fusion gene *mitofusin1*.

**Conclusion:**

An increase in MM was associated with heightened sensitivity of CD4^+^T cells to pyroptosis, even in early differentiated and inactivated CD4^+^T cells, in patients with HIV-1 infection, regardless of whether patients were on antiretroviral therapy or not. These new revelations have uncovered a previously unappreciated challenge to immune reconstitution with antiretroviral therapy.

## Introduction

In HIV-infected individuals, progressive loss of CD4^+^T cells causes AIDS (Acquired Immunodeficiency Syndrome) [1, 2]. After nearly four decades of extensive studies, various mechanisms related to CD4^+^T cell exhaustion have been proposed [3–5]. Among them, apoptosis and pyroptosis have been suggested to be the two main modes of CD4^+^T cell death, especially the latter, which contributes to over 95% of the depletion of CD4^+^T cells during HIV disease progression [6, 7]. What governs this sensitivity to pyroptosis remains poorly understood.

Some studies have indicated a role of caspase-1 in mediating pyroptosis, even in abortive HIV infection [6, 8, 9]. Activated caspase-1 can precipitate mitochondrial disassembly and inhibit mitophagy to amplify mitochondrial damage further, suggesting a link between mitochondria and the pyroptosis process [10, 11]. We recently found that elevated mitochondrial production of reactive oxygen species (ROS), hyperpolarization of mitochondrial membrane potential (MMP) and increased mitochondrial mass (MM) in CD4^+^T cells were concomitant with the progressive loss of CD4^+^T cells during HIV infection. Moreover, the MM was most significantly increased in HIV + patients whose CD4^+^T cell counts were below 200/µL, indicating a possible relationship between the increased MM and CD4^+^T cell exhaustion [12]. Whether mitochondria change and pyroptosis act together to cause CD4^+^T cell depletion in the context of HIV infection may be biologically important.

HIV infection leads to chronic activation of the adaptive immune system, with increased production of pro- and anti-inflammatory cytokines that potentially sensitize CD4^+^T cells to pyroptosis [13–17]. CD4^+^T cells exhibit a skewed maturation from naive (T_N_) and central memory (T_CM_) towards the effector memory cells (T_EM_). This fact raises the question of whether the increased activation and skewed maturation prime CD4^+^T cells for pyroptosis or whether pyroptosis is a predictive factor of this process [15, 16, 18]. Successful highly active antiretroviral therapy (HAART) can result in normal CD4^+^T cell counts and undetectable HIV viremia in most HIV infected individuals [19, 20]. When patients stop taking HAART, however, their CD4^+^T cell counts usually drop rapidly to pretreatment levels [21, 22]. Therefore, it is of medical importance to study carefully the mechanisms of CD4^+^T cell exhaustion in patients with successful virological control. In this study, we examined whether there was a link between mitochondrial change and the sensitivity of CD4^+^T cells to pyroptosis using cells from HIV-1 infected patients who were either HAART naive or HAART treated with undetectable viral load. Our data demonstrated that the mitochondrial network contributed to the susceptibility of CD4^+^T cells to pyroptosis, especially for CD4^+^T cells that were either at early differentiation stages or inactivated, regardless of whether the patients were under HAART or not. Furthermore, an increased level of mitochondrial fusion gene characterizes naive CD4^+^T cells in HIV-infected individuals, and the increased mitochondrial mass correlated with the expression of the mitochondrial fusion gene mitofusin1.

## Participants and methods

### Patients and control participants

Patients from the Outpatient Clinic of Beijing Ditan Hospital, Capital Medical University were enrolled in this study. Written informed consent was obtained from each participant. Patients with chronic diseases, neoplasms, immune inflammatory diseases, other non-HIV-related diseases, and metabolic complications were excluded from this study. A total of 60 HIV-infected men-who-have-sex-with-men (MSM) patients were included: 31 were antiretroviral therapy untreated at the time of enrolment (UNT ) and 29 had been on antiretroviral therapy for more than one year and had undetectable viremia (ART). Twenty-one healthy HIV-negative, age-adjusted MSM donors (HD) were included as controls (Table 1).

**Table 1:** Clinical characteristics of the individuals included in the present study

	Healthy controls	HIV-infected no-ART	HIV-infected ART-exposed
**numbers**	21	31	29
**Sex(males/females)**	21/0	31/0	29/0
**age**	27(23–28)	28(23–33)	30(26–36)
**CD4T cells/ul**	NA	355(17–956)	608(209–1280)
**nadirCD4T cells/ul**	NA	355(17–956)	347(68-1032)
**VL(copies/ml)**	--	68,255(3329-137210)	TND
**ART regimen**	--	--	TDF + 3TC + EFV
**years of ART**	--	--	1–2
**Sexual preference**	male homosexuality	male homosexuality	male homosexuality

### Flow cytometry

Peripheral blood mononuclear cells (PBMCs) were isolated by density centrifugation using Ficoll-Hypaque (Amersham Biosciences, Amersham, Buckinghamshire, UK) from 20 mL venous blood samples collected in EDTA tubes. Freshly isolated PBMCs at 1 × 10^6^ per tube were stained as previously described [12]. The following monoclonal antibodies were used for T cell immunophenotyping: CD3-APC, CD4-APC-CY7, CD45RA-PE-CY7, CCR7-PERCP-CY5.5 and HLADR-V500 (BD Biosciences, San Jose, CA, USA). After antibody staining, the cells were incubated with 200 µL of 100 nM MitoLite™ Orange FM at 37 °C for 15 min, and washed twice with phosphate-buffered saline. After surface staining with antibodies and mitochondrial staining with MitoLite, Caspase 1-FITC was used for intracellular staining. The isotype control of FITC, PE-CY7, V500 and PERCP-CY5.5 was performed for each experiment to determine gates for them.

### Isolation of naive CD4^+^T cells

Naive CD4^+^T cells were enriched by MACS (magnisort cell separation) negative selection using the naive CD4^+^T isolation kit (AutoMACS; Miltenyi Biotec, San Jose, CA, USA). The purity of isolated cells was > 95%, based on assay with CD4-APC-CY7 and CD45RA-PE-CY7 staining and FACS (fluorescence activating cell sorter) analysis.

### RNA isolation and mitochondrial related mRNA detection by qRT-PCR

Total RNA from naive CD4^+^T cells was extracted using the QIAamp RNA Mini Kit (Qiagen, Hilden, Germany). The transcript levels of mitofusin1 (MFN1), mitofusin2 (MFN2), optic atrophy 1 (OPA1) and dynamin-related protein1 (DRP1) were measured using a quantitative real-time reverse transcription PCR (qRT-PCR) assay; β-globin was used as an internal control gene. All the probes and primers used were purchased from Thermo Fisher (Waltham, MA, USA).

### IL-18 and IL-1β measurements

Plasma levels of IL-18 and IL-1β were quantified by the Luminex xMAP technology of R&D Systems (Minneapolis, MN, USA) Luminex Assays (ZLXSAHM-06). In regards to IL-1β, the maximum detection concentration is 14,520 pg/ml and the minimum detection concentration is 1.14pg/ml. For IL-18, the maximum detection concentration is 5461pg/ml and the minimum detection concentration is 3.9pg/ml. All our data falls within these ranges.

### Plasma HIV-1 viral load and CD4^+^T cell count

Plasma HIV-1 RNA levels and CD4^+^T cell counts were measured as described in previous studies twice per year [23], and both measurements were included in the National Quality Assurance Programs.

### Statistical analysis

Group data were expressed as median and interquartile range (IQR) or mean and standard deviation when appropriate. For data that could not be assumed to have a normal distribution, comparisons among several groups were made using a one-way Kruskal-Wallis test (p ≤ 0.05 was considered statistically significant). If the Kruskal-Wallis test indicated significance, the Mann-Whitney test was used for post hoc analysis for comparisons between two groups, with Bonferroni corrections for multiple comparisons, for which P < 0.05 × 2/k (k–1) was considered statistically significant (k refers to the number of groups). The correlations were analyzed with the Spearman’s correlation test.

The above mentioned analyses were performed using either Prism5.0 (GraphPad, La Jolla, CA, USA) or SPSS 13.0 (College Station, TX, USA) software.

## Results

### CD4^+^T cells display a higher level of MM in HIV-infected patients

**Fig. 1:**
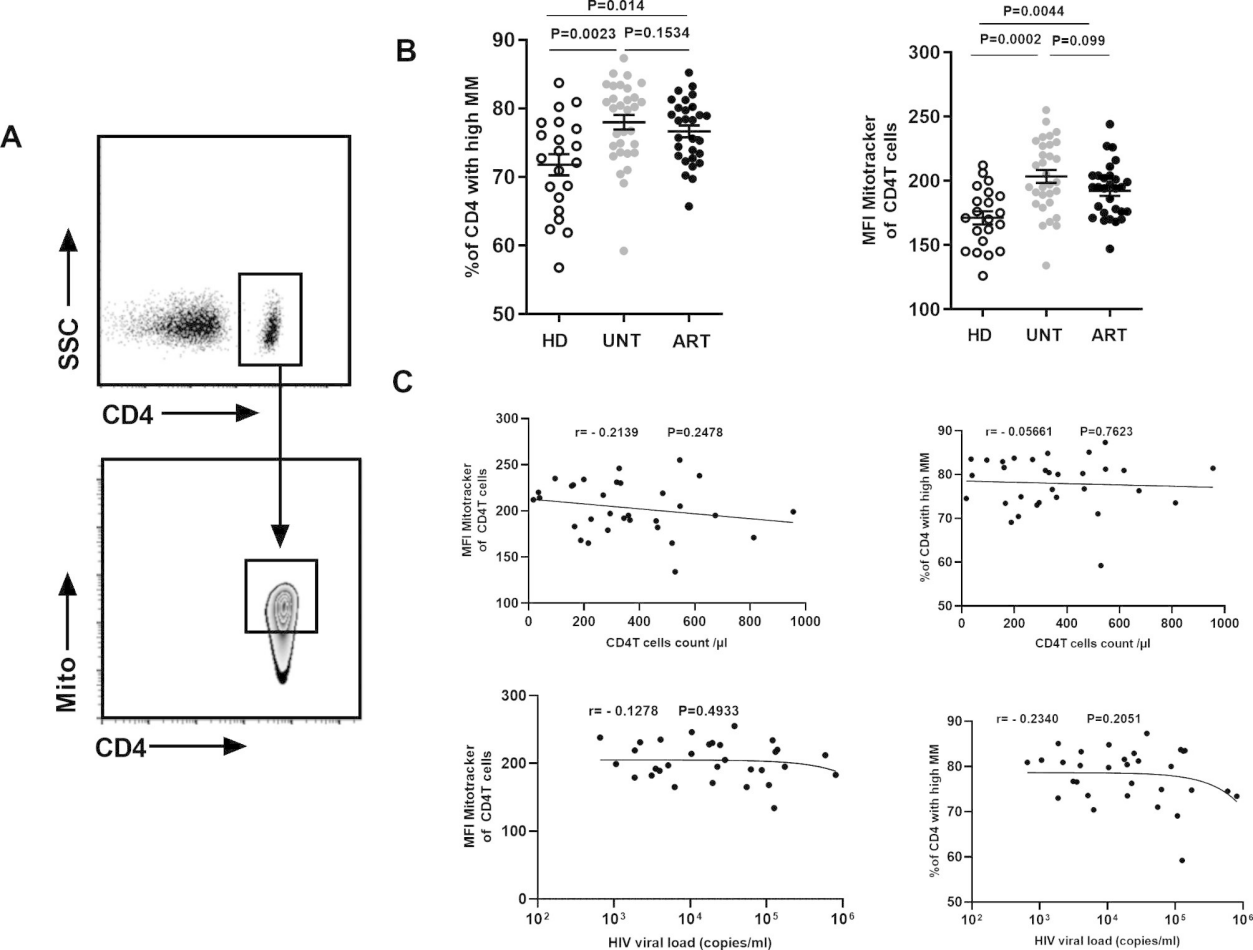
CD4^+^ T cells express higher MM in HIV-infected patients compared with healthy donors. (**A**) The gated CD4^+^T cells were divided into two groups: one exhibiting high MM, the other exhibiting low MM. (**B**) The frequency of MM^high^ CD4^+^T cell subsets and the mean fluorescence intensity of MM in CD4^+^T cells were evaluated in peripheral blood from HD (n = 21), UNT (n = 31), and ART (n = 29). (**C**) The correlation between the MFI of MM in CD4^+^T cells and MM^high^ expression level with viral load and CD4^+^T cells counts in UNT.

To examine the possible involvement of mitochondria in pyroptosis of CD4^+^T cells in HIV infection, we first measured the MM of freshly isolated PBMC from UNT, ART and HD using a mitochondrial specific dye (MitoLite™ Orange FM), which binds the mitochondrial membrane independently of the membrane potential and gives an index of mitochondrial mass based on staining intensity [12, 24]. When gated on the CD4^+^T cells, two distinct populations were divided: one exhibiting higher MM and the other with lower MM (Fig. 1A). Mitichondrial mass high means there is a large amount of mitochondria present in the cells being analyzed. It could indicate an increase in mitochondrial biogenesis (the process of creating new mitochondria) or an accumulation of mitochondria due to cellular stress or dysfunction [11]. The percentage of CD4^+^T from UNT with high MM (78.0%±5.9%, n = 31) was found to be significantly higher than that from HD (71.8 ± 7.0%, n = 21, P = 0.0023) (Fig. 1B). Furthermore, there was a greatly increased mean fluorescence intensity (MFI) of MM, an index of the MM per cell, in CD4^+^T from UNT (MFI = 203.4 ± 28.0) compared with that from HD (MFI = 171.2 ± 23.3, P = 0.0002).

No difference in MM^High^ was found between CD4^+^T cells of ART and UNT (76.7%±4.7% vs. 78.0%±5.9%). ART had significantly higher levels of MM^High^ CD4^+^T cells (76.7%±4.7%, n = 31) than HD (71.8%±7.0%, n = 21, P = 0.014), and increased MFI of MM (MFI = 192.2.4 ± 21.0 vs. MFI = 171.2 ± 23.3, P = 0.0044) (Fig. 1B). Our data revealed that a significant change in MM characterized CD4^+^T cells in HIV-1 infected patients with or without antiretroviral therapy. We did not found there were correlations of percent of CD4^+^T cells with high MM, MFI mitotracker of CD4^+^T cells with viral load and CD4^+^T cell counts.(Fig. 1C).

### MM^high^ CD4^+^T cells from HIV-infected patients have a tendency towards pyroptosis

**Fig. 2:**
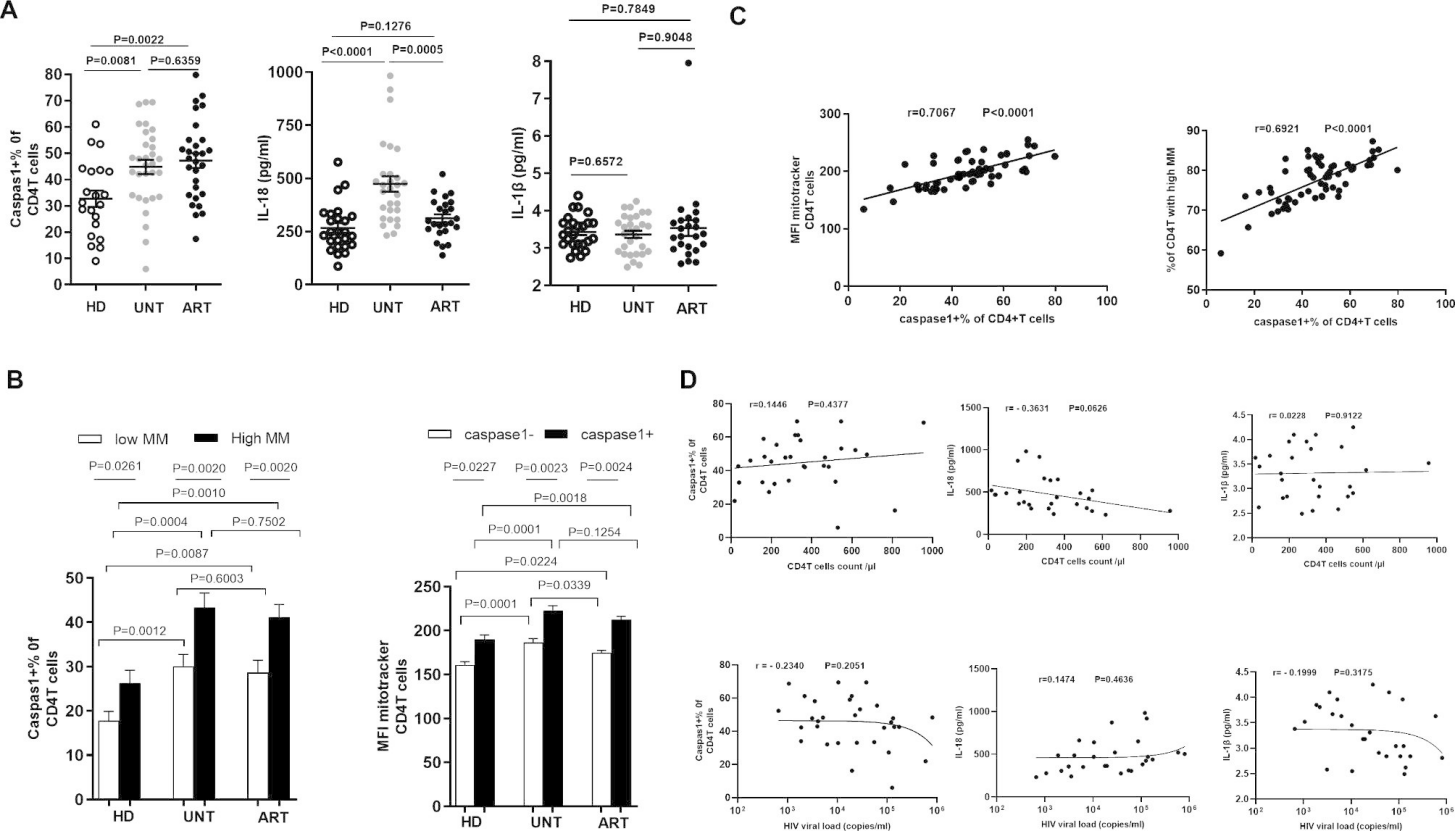
MM^high^ CD4^+^ T cells from HIV patients are predisposed to pyroptosis. (**A**) Percentages of caspase-1(+) in CD4^+^T cells from HD, UNT, ART. IL-18 and IL-1β in plasma from HD, UNT, and ART. (**B**) Pyroptosis was measured in MM^high^ and MM^low^ CD4^+^T cells by caspase-1 staining. Pooled data showing the percentage (%) of caspase-1(+) T cells in MM high and MM low CD4^+^T cells and the MFI of MM in caspase-1(+) CD4^+^T cells and caspase-1(–) CD4^+^T cells. (**C**) The correlation between the frequency of MM^high^ CD4^+^T cells and the percentage of caspase-1(+) CD4^+^T from HIV-infected patients regardless of whether under antiretroviral therapy (n = 60). The correlation between the MFI of MM in CD4^+^T cells and caspase-1 expression level in the context of HIV infection (UNT and ART). (**D**) The correlation between the the level of IL-18, IL-1β and percentages of caspase-1(+) in CD4^+^T cells with viral load and CD4^+^T cell counts in UNT.

Next, we explored whether CD4^+^T cells from HIV-infected patients exhibiting the MM^high^ phenotype were more sensitive to pyroptosis compared with those that were MM^low^. First, measurement of CD4^+^T cell pyroptosis using caspase-1 staining revealed that the ratio of CD4^+^T cells expressing caspase-1 was significantly higher in UNT (n = 31, 44.8%±15.1%) than in HD (n = 21, 32.7%±14.2%, P = 0.0081). No difference in caspase-1 was found between CD4^+^T cells from ART and UNT (47.3%±15.2% vs. 44.8%±15.1%, P = 0.6359). ART had significantly higher levels of caspase-1(+) CD4^+^T cells (n = 31; 47.3%±15.2%) compared with HD (n = 21; 32.7%±14.2%, P = 0.0022). In addition, the expression of IL-18 was significantly higher in UNT (n = 29) than in HD (n = 25) (474.4 ± 36.0 vs. 267.1 ± 22.34, P < 0.0001), and the level of expression decreased to normal after successful suppression of the viremia (n = 24, 312.7 ± 18.9 vs. 267.1 ± 22.34, P = 0.1276). There was no statistically significantly difference in the expression of IL-1β among the three groups (Fig. 2A). We did not find there were correlations of percent of CD4^+^T expressing caspase-1, the levels of IL-18 and IL-1β with viral load and CD4^+^T cell counts.(Fig. 2D).

Moreover, CD4^+^T cells that had undergone caspase-1(+) had a higher MFI MM compared with caspase-1(-) CD4^+^T cells in the three groups (HD: n = 21, 26.4%±13.4% vs. 17.8%±9.4%, P = 0.0277; UNT: n = 31, 43.3%±19.1% vs. 30.1%±15.5%, P = 0.0023; ART: n = 29, 41.2%±15.1% vs. 28.6%±15.1%, P = 0.0024). The difference in MFI MM between caspase-1(-) CD4^+^T cells and caspase-1(+) CD4^+^T cells was more significant in UNT than in HD (P = 0.0023 vs. P = 0.0277). MM^high^ CD4^+^T cells were found to be more sensitive to caspase-1 activated pyroptosis (40.6%±13.3%) compared with MM^low^ cells (28.0%±12.1%) in UNT (Fig. 2B).

Furthermore, a significant correlation was found between the MFI of MM and caspase-1(+) percentage in CD4^+^T cells from HIV patients regardless of whether they were on ART or not (n = 60, r = 0.7067 P < 0.0001). Significant correlation was also observed between the percentage of MM^high^ and caspase-1 in CD4^+^T cells from the two HIV + groups ( UNT and ART ) (r = 0.6921, P < 0.0001) (Fig. 2C). Therefore, our data clearly demonstrated that increased MM was linked to sensitivity of CD4^+^T cells to pyroptosis, and MM^high^ CD4^+^T cells from HIV + groups were more sensitive to pyroptosis than those from healthy controls.

### High expression of HLA-DR alone does not explain increased MM in CD4^+^T cells from HIV + individuals

**Fig. 3:**
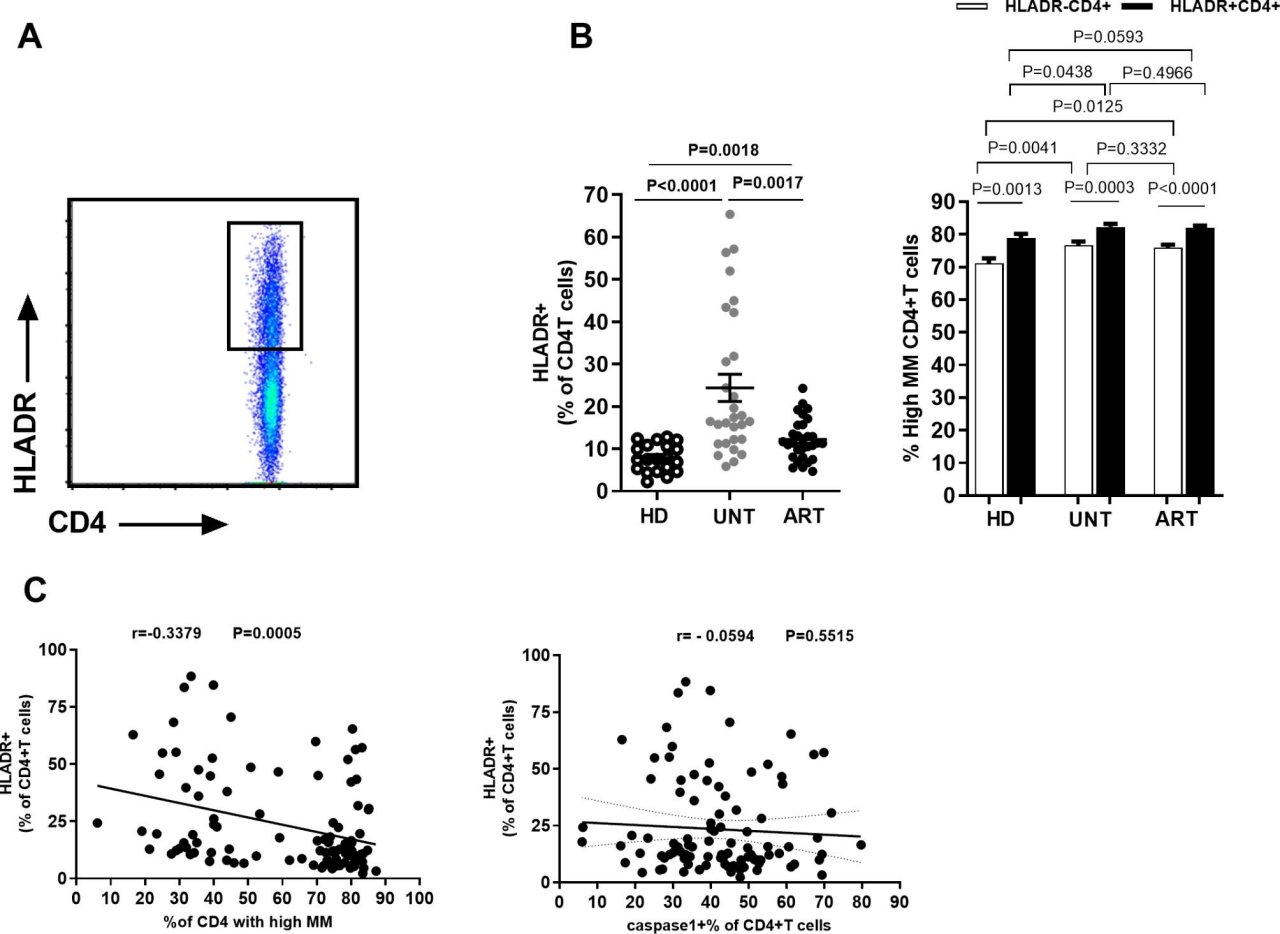
HLA-DR expression, although a negative correlate of MM, does not explain the pyroptosis of CD4^+^ T cells. (**A**) Flow cytometry showing HLA-DR expression gating in CD4^+^T cells. The frequency of HLA-DR(+) CD4^+^T subsets in peripheral blood from HD, UNT and ART. (**B**) MM^high^ percentages were measured in HLA-DR(+) and HLA-DR(–) CD4^+^T cells in peripheral blood from HD, UNT and ART. (**C**) The correlation between the MFI of MM in CD4^+^T cells and HLA-DR expression level in the context of HIV infection (UNT and ART) (r=–0.3317, p = 0.0005). The correlation between the frequency of HLADR expression level and the percentage of caspase-1(+) CD4^+^T from HIV-infected patients regardless of whether under ART (UNT and ART) (n = 60)

Next, we investigated whether the MM of CD4^+^T cells were associated with their activation status (surface expression of the HLA-DR marker). First, we use CD3 antibodies to label T cells, and then CD4 antibodies to label CD4-positive T cells. Then, we further divide CD4-positive T cells into two groups: activated and inactive. We use HLA-DR antibodies to label activated CD4^+^T cells. Finally, expression levels of MM and caspase-1 were detected. A significantly increased percentage of HLA-DR + cells was found among CD4^+^T cells from UNT compared with those from HD (24.4%±17.2% vs. 7.9%±3.3%, P < 0.0001). Although a significantly decreased percentage of HLA-DR + cells was found in CD4^+^T cells from ART compared with UNT (12.2%±4.9% vs. 24.4%±17.2%, P = 0.0017), ART still had a significantly higher percentage of HLA-DR + cells compared with HD (P = 0.0018) (Fig. 3A). MM was then measured in HLA-DR + and HLA-DR– CD4^+^T cells. HLA-DR^+^CD4^+^T cells tended to have a higher MM^high^ percentage compared with HLA-DR^–^CD4^+^T cells in the three groups (HD: n = 21, 78.8 ± 6.0 vs. 71.1 ± 7.1, P = 0.0013; UNT: n = 31, 82.2 ± 5.4 vs. 76.0 ± 4.8, P = 0.0003; ART: n = 29, 82.0 ± 4.1 vs. 76.0 ± 4.8, P < 0.0001). However, the difference in MM level was significant only in HLA-DR^–^CD4^+^T cells, not in HLA-DR^+^CD4^+^T cells, in the context of HIV infection (HLA-DR-: UNT vs. HD P = 0.0041, ART vs. HD P = 0.0125, ART vs. UNT P = 0.3332; HLA-DR + UNT vs. HD P = 0.0438, ART vs. HD P = 0.4966, ART vs. UNT P = 0.0593 ). (Fig. 3B). We found a significant inverse correlation between HLA-DR + percentage and MM^high^ percentage in CD4^+^T cells from HIV-infected patients regardless of whether they were on ART or not (r=-0.3379, P = 0.0005). No correlation was found between HLA-DR + percentage and caspase-1 + percentage in CD4^+^T cells from HIV-infected patients (Fig. 3C). These results demonstrated that the increased HLA-DR + percentage had a low negative effect on the increased MM in CD4^+^T cells, but it did not influence the linkage between increased MM and pyroptosis sensitivity of CD4^+^T cells in HIV-1 patients.

### Increased mitochondrial mass (MM) in CD4^+^T cells is mainly caused by HIV infection but not the disordered differentiation of CD4^+^T cells

**Fig. 4:**
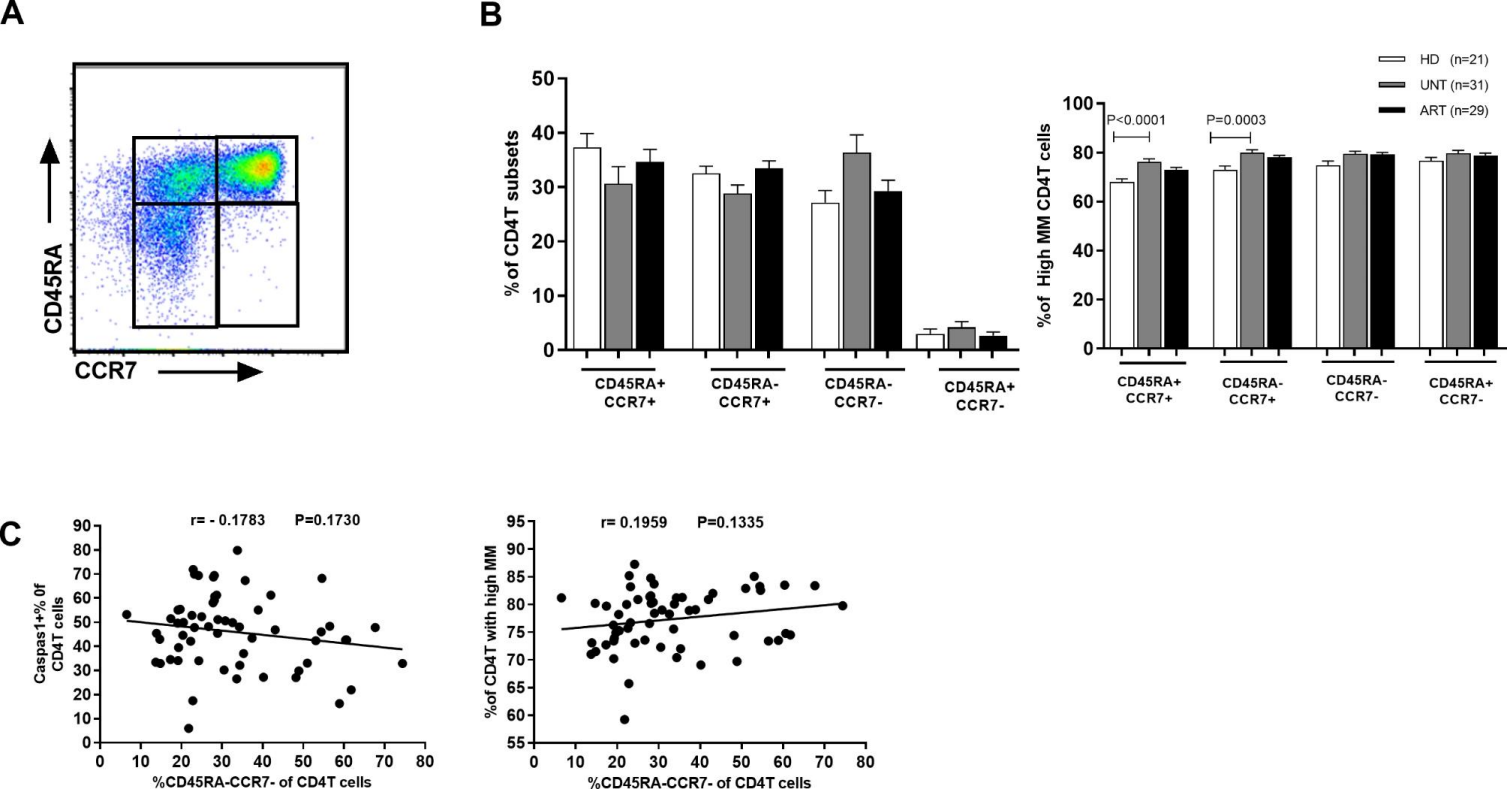
MM expression level in CD4^+^ T cells of different differentiation status. (**A**) Flow cytometry showing naive (CD45RA^+^CCR7^+^), central memory (CD45RA^–^CCR7^+^) and effector memory (CD45RA^–^CCR7^–^) and terminally differentiated cells (CD45RA^+^CCR7^–^) subset gating strategies. The frequency of naive, central memory, and effector memory CD4^+^T subsets in peripheral blood CD4^+^T cells from HD, UNT and ART. (**B**) MM^high^ percentage was measured in naive, central memory and effector memory CD4^+^T subsets in peripheral blood from HD, UNT and ART. Note: Only the p-values indicating statistical differences are marked. (**C**) The correlation between the MFI of MM in CD4^+^T cells and the percentage of effector memory (CD45RA^–^CCR7^–^) CD4^+^T subsets in the context of HIV infection (UNT and ART). The correlation between the frequency of effector memory (CD45RA^–^CCR7^–^) CD4^+^T subsets and the percentage of caspase-1(+) CD4^+^T from HIV-infected patients regardless of whether under ART (n = 60)

The above data have shown that an increased MM was linked to pyroptosis sensitivity of CD4^+^T cells; however, it is unknown whether MM is independent of the differentiation level of CD4^+^T cells. In HIV-1 infection, CD4^+^T cells exhibited a skewed maturation from naive (CD45RA^+^CCR7^+^) and central memory (CD45RA^−^CCR7^+^) toward the effector memory (CD45RA^–^CCR7^–^) compartment [15, 16]. Therefore, the observed differences in MM between caspase-1(+) CD4^+^T cells and caspase-1(-) CD4^+^T cells, at least in part, could be accounted for by their different maturation levels. No significant change was observed in the percentages of naive (CD45RA^+^CCR7^+^) CD4^+^T cells, central memory (CD45RA^–^CCR7^+^), effector memory (CD45RA^–^CCR7^–^), and terminally differentiated cells (CD45RA^+^CCR7^–^) CD4^+^T cells from UNT or ART compared with HD (Fig. 4B). This is agreement with previously published data [25]. Compared to those in HD, higher percentages of MM^high^ were found in naive CD4^+^T cells of UNT (76.2% ± 6.9% vs. 68.0%±6.2%, P < 0.0001), as well in CM CD4 + T cells (80.0%±6.3% vs. 73.0%±7.2%, P = 0.0003). Comparable percentages of MM^high^ effector memory CD4^+^T cells and terminally differentiated CD4^+^T cells were found in UNT vs. HD (P = 0.0177, P = 0.2068) (Fig. 4B). No correlation was found either between the percent of caspase-1 + of CD4^+^T cells and the effector memory CD4^+^T percentage or between the MM of CD4^+^T cells and the effector memory CD4^+^T cell percentage among two HIV-infected gorups (UNT and ART) (Fig. 4C). Although increased MM was most evident in naive CD4^+^T cells, the MM^high^ percentages in three CD4^+^T subsets from UNT were all higher than those from HD. Thus, higher mitochondrial mass (MM) in CD4^+^T cells was mainly associated with HIV infection but not the disordered differentiation of CD4^+^T cells.

### Increased mitochondrial fusion characterizes naive CD4^+^T cells from UNT

Based on the above data, HIV-1 infection led to increased MM of CD4^+^T cells and caspase-1 activated pyroptosis of CD4^+^T cells, especially those CD4^+^T cells of early differentiation status. Therefore, we next examined whether the mitochondrial fission and fusion balance was altered in naive CD4^+^T cells and, if so, how it contributed to the increased MM of CD4^+^T cells in HIV-1 infection. Mitochondrial fission is mediated by a dynamin family member, DRP1 (dynamin-related protein 1). Mitochondrial fusion between mitochondrial outer membranes is mediated by MFN1 and MFN2, whereas fusion between mitochondrial inner membranes is mediated by OPA1 (optic atrophy protein 1) [26–28]. After isolation of the naive CD4^+^T cells from UNT, ART and HD, we measured the levels of expression of DRP1 mRNA, MFN1 mRNA, MFN2 mRNA, and OPA1 mRNA.

**Fig. 5:**
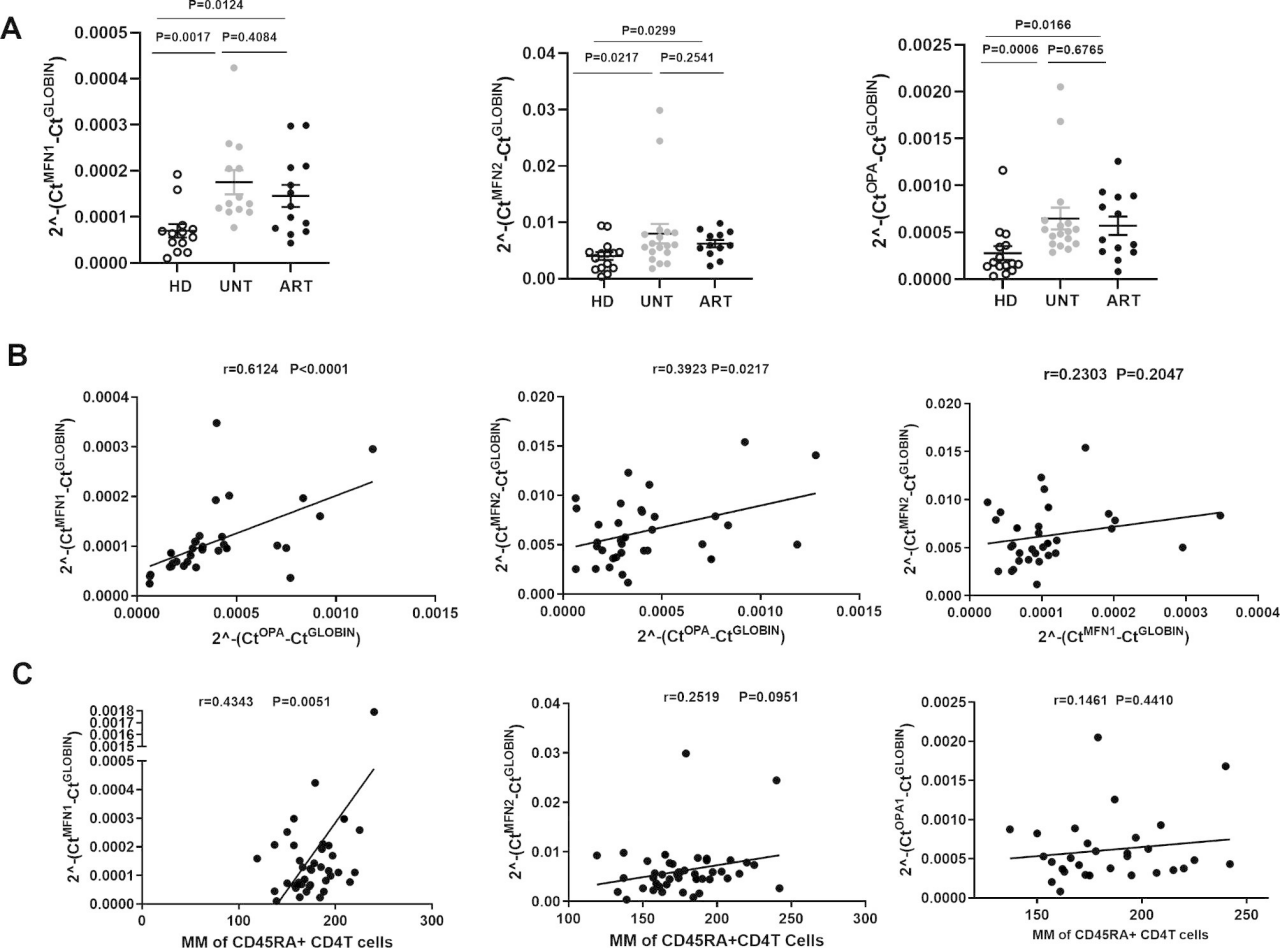
Increased mitochondrial fusion characterizes naive CD4^+^ T cells from HIV-1 infected patients, and MFN1 is related to the increased MM. (**A**) Significantly increased MFN1, MFN2 and OPA1 expression in naive CD4^+^T cells was found in the HD, UNT and ART. (**B**) The correlations among the levels of mRNA expression of three mitochondrial fusion genes (MFN1, MFN2, and OPA1). (**C**) Significant correlation was found between the MFI of MM and MFN1 expression in naive CD4^+^T cells from HIV patients regardless of whether under ART (n = 28), but this was not found for MFN2 and OPA1.

A significantly increased expression of MFN1, MFN2 and OPA1 in naive CD4^+^T cells was found in UNT compared with HD (MFN1: P = 0.0017; MFN2: P = 0.0217; OPA1: P = 0.0006), suggesting an increased mitochondrial fusion of naive CD4^+^T cells in HIV infection. We did not detect DRP1 expression in CD4^+^T cells from three groups. No differences in MFN1, MFN2 and OPA1 expression were found between naive CD4^+^T cells from ART (n = 13) and those from UNT (n = 14). ART still had significantly higher levels of MFN1, MFN2 and OPA1 expression compared with HD (MFN1: P = 0.0124; MFN2: P = 0.0299; OPA1: P = 0.0166) (Fig. 5A). Correlations between the expression of these genes were also analyzed among UND and ART (n = 27) as shown is Fig. 5B. Our data revealed that increased mitochondrial fusion characterized naive CD4^+^T cells from HIV-1 infected patients regardless of whether they were on ART regimens.

### High expression of MFN1 is related to an increased mitochondrial mass of naive CD4^+^T cells in patients with HIV-1 infection

A significant correlation was found between the MFI of MM and MFN1 expression in naive CD4^+^T cells obtained from HIV-infected patients regardless of whether they were on antiretroviral therapy or not (n = 28, r = 0.4343 P = 0.0051) (Fig. 5C). In contrast, no significant correlation was observed between the percentages of MM and MFN2 (OPA1) expression. Our data indicated that increased mitochondrial fusion contributed to the increased MM, in association with an elevated expression of the MFN1 gene. We did not find a correlation between caspase-1 levels and MFN1 expression (data not shown).

## Discussion

Recently, increasing numbers of studies have demonstrated that mitochondria had an important role in the differentiation, activation, and death pathways of immune cells [29, 30]. Meanwhile, whether mitochondrial toxicity is the mechanism underlying the exhaustion of CD4^+^T cells has also received some attention in the context of HIV infection [12, 31, 32]. Based on our previous findings that mitochondrial mass (MM) in CD4^+^T cells gradually increases in association with the progressive loss of CD4^+^T cells during HIV infection [12], and that pyroptosis accounts for 90% of CD4^+^T cell death [6], we hypothesized that the increased MM in CD4^+^T cells may be related to their susceptibility to pyroptosis, and tested the hypothesis by using MitoLite TM Orange FM as an index of MM [33] and caspase-1 staining for pyroptosis [6]. We found that MM was significantly increased in caspase-1 activated CD4^+^T cells from HIV-infected patients, indicating that the MM^high^ phenotype specifically characterized pyroptosis of CD4^+^T cells. The MM^high^ phenotype of CD4^+^T cells could not be attributed to disordered CD4^+^T cell differentiation status, as similar levels of MM were found in CD45RA^+^CCR7^+^, CD45RA^–^CCR7^+^, and CD45RA^–^CCR7– in CD4^+^T cells from UNT, indicating that this mitochondrial parameter was independent of the disordered differentiation status often present in HIV infection. Meanwhile, over-activation could not explain the relationship between an increased MM and a susceptibility to pyroptosis because we did not find significant correlation between HLA-DR expression and MM level, or the percentage of caspase-1 expression.

In this study, we have demonstrated that MM^high^ CD4^+^T cells were sensitive to pyroptosis, directly linking mitochondria to the pyroptosis sensitivity of CD4^+^T cells. This phenomenon should be more significant in immunological non-responders because the highest MM was observed in patients whose CD4^+^T cell count was below 200/µL [12]. Owing to the small sample size, we did not analyze our current data based on CD4 cell number strata. Our previous large-scale study clearly confirmed the presence of mitochondrial toxicity in CD4^+^T cells in HIV-infected patients [12].

Based on previous study demonstrating that activated caspase-1 precipitates mitochondrial disassembly and inhibits mitophagy to amplify mitochondrial damage, we hypothesized that the increased MM, as a result of either larger or more organelles, intensified the damage in mitochondria, and further enhanced pyroptosis of CD4^+^T cells in HIV infection. We could not distinguish whether high MM corresponded to larger organelles or higher numbers by the flow-cytometry-based assay, as both would result in higher levels of staining. Therefore, the expression of mitochondrial fusion and fission genes was measured, because excess mitochondrial fusion leads to larger mitochondria and heightened mitochondrial fission leads to higher numbers [26, 33]. Increased mitochondrial fusion genes (MFN1, MFN2, OPA1) were found in naive CD4^+^T cells from HIV-infected individuals, compared with healthy controls, indicating that excessive mitochondrial fusion may exist in CD4^+^T cells during HIV infection. This may be one of the mechanisms responsible for the increased MM-related pyroptosis, because excess mitochondrial fusion contributes to an accumulation of damaged mitochondria. More importantly, we found a significant correlation between MFN1 gene expression and MM levels, indicating that the MFN1 gene may play an important role in MM accumulation in CD4^+^T cells. Although no significant correlation was found between caspase-1 and MFN1 expression, we cannot rule out that overexpression of MFN1 may be related to the susceptibility to pyroptosis in CD4^+^T cells. Further study is needed to explore how HIV infection influences the expression of the MFN1 gene, and whether the MFN1 gene encodes an important signal molecule in the pathway of pyroptosis in CD4^+^T cells. We did not detect the expression of the mitochondrial fission gene DRP1 in peripheral blood CD4^+^T cells from either HIV-infected individuals or healthy donors. Mitochondrial fusion can maximize oxidative capacity in response to toxic stress and mitigate environmental damage through the exchange of proteins and lipids with other mitochondria [26, 28, 33]. However, when a certain threshold of damage is reached, increased fusion may lead to cell death because of a lack of renewal [33, 34]. Therefore, further study is needed to explore how the increased MM affects caspase-1 activated pyroptosis of CD4^+^T cells in HIV-1 infection, and if the excessive fusion has an effect on it.

As the mitochondrial toxicity of HAART drugs develops in patients treated for more than 2 years [12], patients who were treated for 1–2 years were recruited to represent the effects of chronic HIV infection in this study. Successful HAART can restore CD4^+^T cell counts and lead to undetectable HIV viremia in most HIV-infected individuals [35, 36]. Unfortunately, non-AIDS-defining events such as cardiovascular disease (CVD) and non-AIDS-defining malignancies are still more prevalent in HAART-treated HIV-infected adults than in uninfected adults [37–39], and persistent immune activation and chronic inflammation are often not identified as the mechanism underlying this phenomenon [40–43]. In this study, we found an obvious pathological change (increased of MM and pyroptosis sensitivity) in CD4^+^T cells from HAART-treated patients whose viremia was effectively suppressed, which provides a new pathway to explain the clinical phenomenon. As antiretroviral therapy alone is not sufficient to restore all immunological functions in HIV-infected patients, paying attention to improving the mitochondrial function and ameliorating the susceptibility to pyroptosis of CD4^+^T cells may provide new therapeutic targets. There are some limitations to our study. Since we solely examined patients’ blood, we did not investigate how mfn1 gene knockdown or overexpression affects pyroptosis levels in CD4 + T cells. To determine the impact of mfn1 on CD4 + T cell depletion, further research is required using ex vivo experiments.

## Conclusion

This study suggested that increase in MM was associated with heightened sensitivity of CD4^+^T cells to pyroptosis, even in early differentiated and inactivated CD4^+^T cells, in patients with HIV-1 infection, regardless of whether the patients were on HAART or not. These new revelations have uncovered a previously unappreciated challenge to immune reconstitution with antiretroviral therapy. Further detailed studies of the role of mitochondrial disturbance in the exhaustion of CD4^+^T cells should be performed.

## Data Availability

The datasets generated and/or analysed during the current study are available from the corresponding author on reasonable request.

## List of Abbreviations

HIV: Human immunodeficiency virus
HAART: Highly active antiretroviral therapy
ART: Antiretroviral therapy
MM: Mitochondrial mass
PBMC: Peripheral blood mononuclear cells
CD: Cluster of differentiation
MSM: Men-who-have-sex-with-men
MACS: Magnisort cell separation
FACS: Fluorescence activating cell sorter
HLA-DR: Human leukocyte antigen-DR
IL: Interleukin
CM: Central memory
EM: Effector memory
DRP1: Dynamin-related protein 1
MFN: Mitofusin
OPA1: Optic atrophy protein 1
CVD: Cardiovascular disease
